# A novel agent targeting APRIL: A new hope for elderly patients of IgA nephropathy

**DOI:** 10.1002/agm2.12370

**Published:** 2025-01-20

**Authors:** Xin Liu, Li Zuo

**Affiliations:** ^1^ Department of Nephrology, Beijing Hospital, National Center of Gerontology; Institute of Geriatric Medicine Chinese Academy of Medical Sciences Beijing China; ^2^ Department of Nephrology Peking University People's Hospital Beijing China

**Keywords:** APRIL, elderly, IgAN

## Abstract

Sibeprenlimab is a humanized IgG2 monoclonal antibody that binds to and neutralizes the activity of APRIL. It should usher in a new era in the treatment of IgAN, especially in elderly patients.

## 1 BACKGROUND

Immunoglobulin A nephropathy (IgAN) is the most common primary glomerulonephritis worldwide, characterized by mesangial IgA deposition. Asymptomatic hematuria with varying degrees of proteinuria is the most common clinical presentation, and 20%–40% of patients progress to end‐stage kidney disease within 20 years after disease onset.^1^ Currently, the “four‐hit hypothesis” explains the pathogenesis of IgAN.^2^ This hypothesis suggests that IgAN begins with elevated levels of circulating abnormally glycosylated galactose‐deficient IgA1 (gd‐IgA1), followed by the formation of immune complexes with anti‐gd‐IgA1 antibodies that ultimately deposit in the glomerular mesangium, leading to kidney injury. With the progressive studies of the pathogenesis of IgAN, key molecules in the pathogenesis have gradually become potential targets for future treatment strategies, and corresponding new drugs have been constantly emerging, as shown in Table 1. A proliferation inducing ligand (APRIL) is the 13th member of the TNF superfamily (TNFSF13), and as one of the growth factors of B cells, it can participate in the occurrence and development of IgAN by promoting B cell activation and the generation of gd‐IgA1.^3^ The analysis of genome‐wide association studies further suggests that TNFSF13 is a genome‐wide locus significantly associated with IgAN, identifying the pathogenic signaling pathway and providing important evidence support for targeted drug development.^4^ Blocking APRIL activity is a potential therapeutic approach to reduce circulating levels of gd‐IgA1 and its associated immune complexes.

**TABLE 1:** Novel drugs in clinical trials for IgAN.

Drug	Target	Phase	NCT
Blisibimod	Inhibitor of BAFF	II/III	NCT02062684
Zigakibart	Humanized IgG4 against APRIL	I/II	NCT03945318
Telitacicept	Dual inhibitor of BAFF/APRIL	III	NCT05799287
Atacicept	Antagonist of BAFF/APRIL	II/III	NCT04716231
Felzartamab	Humanized IgG1 monoclonal antibody against CD38	IIa	NCT05065970
Fostamatinib	Spleen tyrosine kinase inhibitor	II	NCT02112838
Pegcetacoplan	Complement C3 inhibitor	II	NCT03453619
Iptacopan	Inhibitor of complement factor B	III	NCT04578834
Narsoplimab	Humanized MASP2 monoclonal antibody	III	NCT03608033
Ravulizumab	Humanized anti‐C5 monoclonal antibody	II	NCT04564339
Vemircopan	Factor D inhibitor	II	NCT05097989

## 2 CURRENT TREATMENT STRATEGIES

The traditional treatment strategy of IgA nephropathy is mainly based on supportive treatment, combined with immunosuppressive therapy. In recent years, great progress has been made in the pathogenesis of IgA nephropathy, and put forward a new viewpoint on the treatment of IgA nephropathy. The new view is that the treatment needs to manage the two basic drivers of continuous nephron loss in IgAN at the same time. The focus of management in most patients should be to prevent or reduce IgA immune complex formation and immune complex‐mediated glomerular injury. In parallel, manage the consequences of existing IgAN‐induced nephron loss.

### 2.1 Optimal supportive care for IgAN

Supportive care mainly delays the loss of glomerular function by reducing glomerular filtration pressure and urinary protein, mainly including strict control of blood pressure, salt restriction, tolerated renin‐angiotensin‐aldosterone system inhibitors (RAASi), sodium glucose cotransporter 2 inhibition (SGLT2i),^5^ Dual endothelin receptor and angiotensin receptor antagonist (Sparsentan),^6^ etc., especially for IgA nephropathy patients with glomerular hyperfiltration caused by chronic renal disease.

### 2.2 Traditional immunosuppressive or immunomodulatory therapy for IgAN

Based on the pathogenesis of IgAN, immunosuppressive therapy is an important approach for treating high‐risk progressive IgAN. However, the use of steroids remains controversial, with inconsistent results from two large multicenter clinical trials, including the TESTING study^7^ and the Supportive versus Immunosuppressive Therapy for the Treatment of Progressive IgA Nephropathy (STOP‐IgAN) study.^8^ Full‐dose steroids often have severe side‐effects, including severe infections. Although the second phase of the TESTING study adopted the reduced glucocorticoid regimen combined with antibiotic prophylaxis for pneumocystis pneumonia to prevent infection, the results showed that there were still a certain proportion of serious adverse reactions and serious infections in the treatment regimen.^7^ For immunosuppressants, most studies have not found them to be effective in treating IgAN. The immunosuppressants, including cyclophosphamide, azathioprine, and calcineurin inhibitors, are not recommended.^9^, ^10^ However, in cases of rapidly progressive glomerulonephritis (RPGN), high‐dose steroids combined with cyclophosphamide may be considered.^11^ In addition, mycophenolate mofetil (MMF) can be used as an alternative option to reduce steroid dosage in Chinese patients with IgAN.^12^ Hydroxychloroquine (HCQ), a classical antimalarial drug, with immunomodulatory and anti‐inflammatory characteristics, has also only been recommended for the treatment of Chinese patients with IgAN.^13^

### 2.3 Intestinal mucosal B cell immunomodulator for IgAN

Nefecon, an enteric‐coated oral formulation of budesonide, partially inhibits the production of pathogenic IgA1 targeted to the Peyer's patches in the ileum.^14^ Phase III clinical trial (NefIgArd study) has shown its efficacy.^15^ As there is some systemic absorption of budesonide patients and clinicians should be aware of the possibility of some systemic glucocorticoid related side effects with nefecon.

## 3 NOVEL AGENT: SPECIFIC ANTIBODIES TARGETING APRIL

Sibeprenlimab (VIS649) is a humanized IgG2 monoclonal antibody that binds to and neutralizes the activity of APRIL. Blocking APRIL activity presents a potential method of treatment to reduce circulating levels of galactose‐deficient IgA1 and associated immune complexes. The Phase II multicenter, double‐blind, randomized, placebo‐controlled trial of sibeprenlimab aims to evaluate the efficacy and safety of the drug in treating adult patients, who had biopsy‐confirmed IgAN.^16^ Eligible patients with estimated glomerular filtration rate (eGFR) ≥30 mL per minute per 1.73 m^2^ (as calculated with the use of the 2009 Chronic Kidney Disease Epidemiology Collaboration equation) and 24‐h urinary protein‐to‐creatinine ratio of at least 0.75 g of protein per gram of creatinine (or a urinary protein level of ≥1.0 g per day), receiving optimal supportive care, were randomized in a 1:1:1:1 ratio to receive intravenous infusion of sibeprenlimab at a dose of 2, 4, or 8 mg per kg of body weight or placebo, once a month for 12 months. The primary efficacy end point was the change from baseline in the 24‐h urinary protein‐to‐creatinine ratio (measured on the natural log scale and derived from a 24‐h urine collection) at month 12. Secondary efficacy end point mainly included the change from baseline in the eGFR at month 12.

A total of 155 patients were randomly assigned to receive sibeprenlimab at a dose of 2 mg per kg (38 patients), 4 mg per kg (41 patients), or 8 mg per kg (38 patients) or placebo (38 patients), with a median follow‐up of 16.0 months. From baseline to month 12, the geometric mean ratio reduction (±SE) in the 24‐h urinary protein‐to‐creatinine ratio 47.2 ± 8.2% in the sibeprenlimab 2‐mg group, 58.8 ± 6.1% in the sibeprenlimab 4‐mg group, 62.0 ± 5.7% in the sibeprenlimab 8‐mg group, and 20.0 ± 12.6% in the placebo group. The least‐squares mean (±SE) changes from baseline in eGFR at the end of the 12‐month treatment period were − 2.7 ± 1.8, 0.2 ± 1.7, and − 1.5 ± 1.8 mL per minute per 1.73 m^2^ in the sibeprenlimab 2‐mg, 4‐mg, and 8‐mg groups, respectively. The average lymphocyte count showed no significant change at month 12 compared to baseline. Adverse events (AEs) were similar across all groups and were mostly mild to moderate in severity. The most common AEs (incidence of ≥5% in the pooled sibeprenlimab group) were coronavirus disease 2019 (COVID‐19), pyrexia, nasopharyngitis, upper respiratory tract infection, headache, hypertension, diarrhea, and muscle spasm. Longer‐term data should be required to assess the safety of targeting B cells and plasma cells by this approach, and any potential impact on immunogenicity.

This Phase II clinical trial demonstrates that sibeprenlimab can significantly reduce urine protein in IgAN patients in a dose‐dependent manner. Limitations of the trial include the relatively short follow‐up period, which precludes the evaluation of the long‐term renal function efficacy of sibeprenlimab, and some confounding factors between groups, such as the slightly younger age, higher proportion of females, higher median baseline eGFR, higher levels of proteinuria, higher crescent formation ratio, and longer biopsy time in the placebo group. The efficacy and safety of sibeprenlimab in a larger population of patients with IgA nephropathy are under investigation in an ongoing phase III trial (VISIONARY; NCT05248646).

## 4 CONCLUSION

In the past, the main treatment strategies for IgAN, especially high‐risk progressive IgAN, included adequate supportive care and glucocorticoid. However, glucocorticoids have more side‐effects, especially in elderly patients, which seriously affect the clinical prognosis of elderly patients. With the advances in the understanding of the pathogenesis of IgAN, the treatment of IgAN is gradually shifting towards towards the level of etiological treatment. Sibeprenlimab, as described above, targets and neutralizes human APRIL at specific antigenic epitopes, inhibiting APRIL‐mediated B‐cell activation and reducing the production of pathogenic IgA1. This novel agent should avoid the use of glucocorticoid and corresponding severe side‐effects, which should usher in a new era in the treatment of IgAN, especially in elderly patients.

## AUTHOR CONTRIBUTIONS

Dr. Liu conceptualized the manuscript, did the literature search and wrote the manuscript draft. Dr. Zuo critically revised the manuscript draft.

## FUNDING INFORMATION

This work was supported by funding program(Beijing Hospital Clinical Research 121 Project:BJ‐2019‐197).

## CONFLICT OF INTEREST STATEMENT

The authors have nothing to report.
